# Supplementary material for: A New Synthetic Curcuminoid Displays Antitumor Activities in Metastasized Melanoma
Source: Cells. 2023 Nov 13;12(22):2619. doi: 10.3390/cells12222619 (PMC10670708; doi:10.3390/cells12222619)
Supplement: Supplementary file 1 [file cells-12-02619-s001.zip › cells-2707830-supplementary.pdf]

Supplementary Materials

A New Synthetic Curcuminoid Displays Antitumor Activities in Metastasized Melanoma

Leonard Kaps [1,*,†], Adrian Klefenz [1,†], Henry Traenckner [1], Paul Schneider [1], Ion Andronache [2], Rainer Schobert [3], Bernhard Biersack [3,*] and Detlef Schuppan [1,4,*]

[1] Institute of Translational Immunology, University Medical Center, Johannes Gutenberg University Mainz, Germany; leonard.kaps@unimedizin-mainz.de, detlef.schuppan@unimedizin-mainz.de

[2] Research Center for Integrated Analysis and Territorial Management, University of Bucharest, Romania; ion.andronache@geo.unibuc.ro

[3] Organic Chemistry 1, University Bayreuth, Germany; rainer.schobert@uni-bayreuth.de, bernhard.biersack@yahoo.com

[4] Division of Gastroenterology, Beth Israel Deaconess Medical Center, Harvard Medical School, Boston, MA, USA

* Correspondence: leonard.kaps@unimedizin-mainz.de (L. K.), bernhard.biersack@yahoo.com (B. B.), detlef.schuppan@unimedizin-mainz.de (D. S.)

† These authors contributed equally to this work.

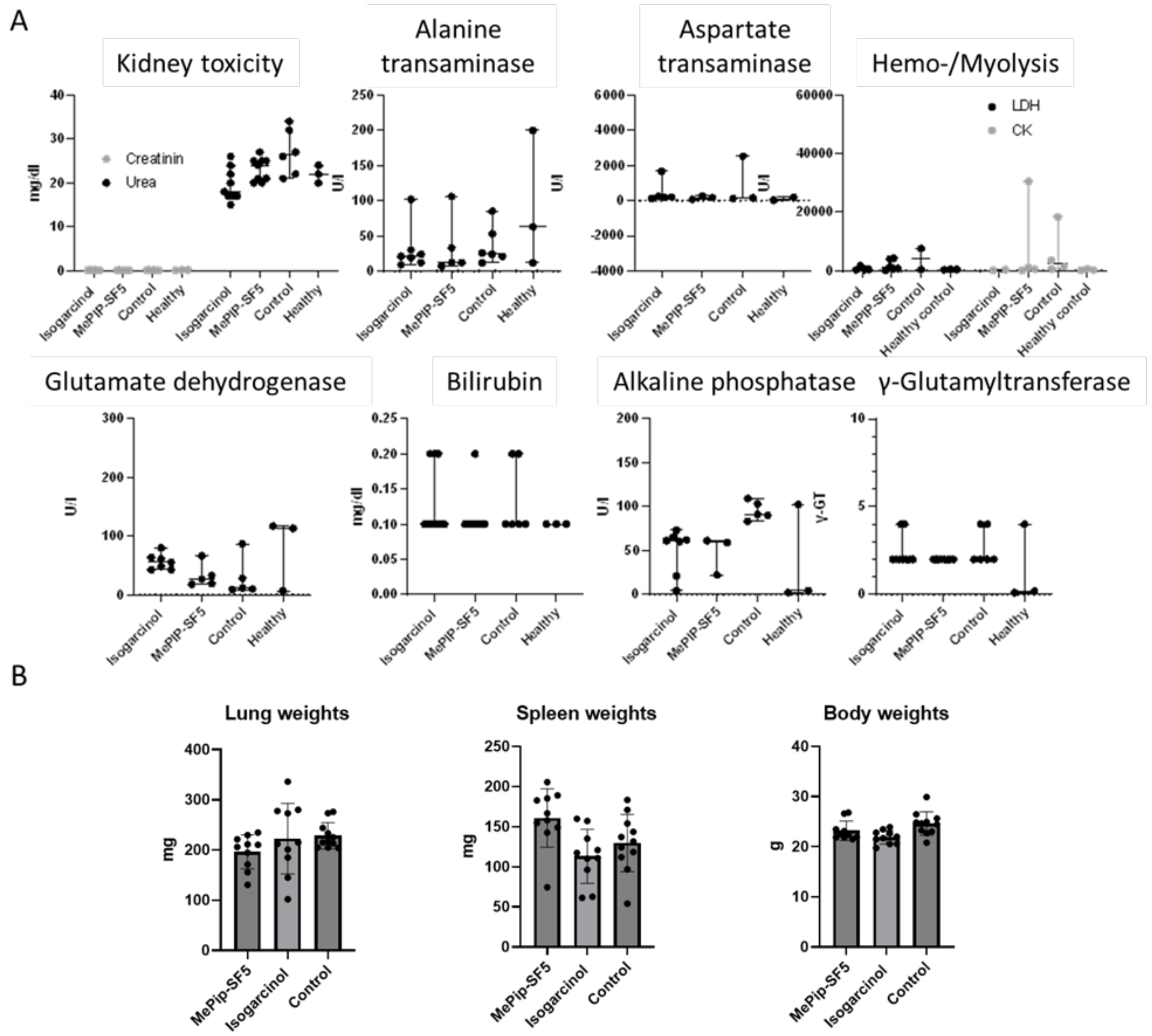

Figure S1. (**A**) Safety laboratory parameters of sera from (un)treated and healthy mice; (**B**) Biometric data of mice at sacrifice.
